# Modeling and interpreting the COVID-19 intervention strategy of China: A human mobility view

**DOI:** 10.1371/journal.pone.0242761

**Published:** 2020-11-24

**Authors:** Haonan Chen, Jing He, Wenhui Song, Lianchao Wang, Jiabao Wang, Yijin Chen

**Affiliations:** 1 College of Geoscience and Surveying Engineering, China University of Mining and Technology-Beijing, Beijing, China; 2 School of Journalism and Communication, Tsinghua University, Beijing, China; Universitat Rovira i Virgili, SPAIN

## Abstract

The Coronavirus Disease 2019 (COVID-19) has proved a globally prevalent outbreak since December 2019. As a focused country to alleviate the epidemic impact, China implemented a range of public health interventions to prevent the disease from further transmission, including the pandemic lockdown in Wuhan and other cities. This paper establishes China’s mobility network by a flight dataset and proposes a model without epidemiological parameters to indicate the spread risks through the network, which is termed as epidemic strength. By simply adjusting an intervention parameter, traffic volumes under different travel-restriction levels can be simulated to analyze how the containment strategy can mitigate the virus dissemination through traffic. This approach is successfully applied to a network of Chinese provinces and the epidemic strength is smoothly interpreted by flow maps. Through this node-to-node interpretation of transmission risks, both overall and detailed epidemic hazards are properly analyzed, which can provide valuable intervention advice during public health emergencies.

## 1. Introduction

The COVID-19 disease has spread worldwide and severely damaged the global economy and public health. Globalization has brought huge traffic volume hence stronger connectivity, which poses serious challenges in the face of such infectious disease with high epidemic potential. Since the first detection and rapid spread of COVID-19 in Wuhan, Hubei province, China has imposed mobility restrictions on Wuhan in January 23, 2020, and sequentially 15 other cities in Hubei province. This lockdown strategy is considered as a key measure to keep the disease from more serious spread. In understanding the dynamics and features of COVID-19, quite a few researchers have focused on the epidemiological factors, such as reproductive number [1, 2], age structure [3, 4] and sex factors [5, 6]. Some other works have concentrated on the estimation of epidemic growth or peak values [7–9]. Additionally, human mobility makes a great difference in discovering transmission dynamics [10]. Brockmann et al. [11] first proposed the concept of effective distance to explore the hidden spatiotemporal patterns in contagion phenomena such as infectious disease. This concept considers the passenger exchange volume between nodes rather can any epidemiological factors, which serves as a robust signal for network-driven dynamic processes of epidemics. Following this idea, Nah et al. [12] regarded arrival time and reciprocal effective distance as the indicator for MERS risk prediction. Similar analysis was conducted on Zika importation risk estimation [13]. Haider et al. [14] suggested a comparable indicator, risk index, that consider passenger import volume and infection rate from other infected nodes. Besides, a range of research works sought to uncover correlations between human mobility and COVID-19 epidemics. For example, Jin et al. [15] studied the relation between migration population from Wuhan and detected coronavirus cases. Kraemer et al. [6] analyzed that China’s movement restrictions can reduce the synchrony between incidence in Wuhan and all other provinces. These works were based on the Baidu migration index, which indicates relative migration flows between Chinese cities other than real traffic volume. To understand how the intervention strategies can affect epidemiological risks of COVID-19 disease, three key issues should be addressed:

1. To establish a human mobility network which represents traveler exchange.
2. To estimate the human mobility in various context during an epidemic or pandemic period.
3. To quantitatively analyze the potential transmission risk involved in the passenger flows.

In this regard, this paper does not discover epidemic patterns by directly analyzing Coronavirus historical data. Instead, our approach estimates the traffic flows during an epidemic period and seeks to measure the epidemic strength, an metric we proposed to indicate the dissemination risk involved in passenger flows.

## 2. Data and methods

### 2.1 Data and mobility network

In this work, we consider a mobility network according to OpenFlights airport database [16]. We extracted the airport and route data in cities across China that covers 185 airports and 1515 routes to construct a human mobility network, where each node denotes an airport and each edge denotes flight routes (S1 Fig). We referred to Mao et al.’s airline passenger model [17, 18] and extracted their monthly data as the normal passenger estimation. The real flight statistics is obtained from monthly reports by Civil Aviation Administration of China (CAAC) [19]. All the coronavirus statistics data are based on Johns Hopkins University (JHU) cases dataset [20] and WHO situation reports [21], of which the recorded period covers from January 22, 2020 to April 16, 2020. We collected population data from World Bank Open Data [22] and the National Bureau of Statistics of China (NBS) [23], whose statistical unit meets provincial grade. We employ subnational administrative boundaries data from the Humanitarian Data Exchange [24] and Earth at Night satellite images from NASA Earth Observatory [25] for map production.

### 2.2 Mobility simulator for pandemic periods

Air traffic estimation involves complexity from many factors, such as historical passenger volume, airport location, local population, economic factors, etc. Due to COVID-19 pandemic situation, the airline traffic can be dramatically damaged thus the simulation incorporates more components. According to Air China Limited’s operational report [26], compared with the market performance last year, the number of monthly carried passengers from February, 2020 to April, 2020 all fell by more than 60%; especially in February, the traffic dropped by 82.9%. Further, in response to the epidemic issue, many scholars and governors suggest cutting down the traffic volume. In this context, we formulate an adjustable model for proper air traffic simulation during epidemic periods, where two fundamental factors are considered: importation risk and intervention impact.

During the early stage of COVID-19, due to the most confirmed cases in China, Haider et al.’s work only considered imported cases from China and weighted their risk estimation with the infected cases in China [14]. The proposed risk index *R*_*n*_ of a destination node *n* can be derived as follows:

$$R_{n}=\sum_{m=1}^{M}\frac{V_{mn}}{V_{m}}∙\frac{I_{m}}{P_{m}}$$ (1)

where *m*∈*M* indicates the origin node, *V*_*mn*_ is the traffic volume from node *m* to node *n*, *V*_*m*_ is the total export volume from node *m*, *I*_*m*_ denotes the infected cases in node *m*, and *P*_*m*_ denotes the total population of node *m*. The metric indicates the importation risk of a certain node to some extent. In a similar but more comprehensive way, we extend this concept to a global model that considers effect from every node in the network, so that the improved model can depict transmission risks in a more generic and robust manner.

Assuming that the mobility network consists of *i* nodes, we compute an *i*-sized set of risk index *R*_*n*_ for each node *n* and compose a shrinkage matrix A for traffic volume restriction. During a period of time *t*, this shrinkage matrix is derived as:

$$A=diag\left(\frac{j-c}{j+c}e^{-R}\right)$$ (2)

where diag(∙) denotes a diagonal matrix created by the target vector input, *j* indicates an *i*-sized vector of ones, *R* = {*R*_1_,*R*_2_,…,*R*_*i*_}∈[0,+∞) is a set of risk index for each node. Corresponding to each node, *c*_*i*_ in *c* = {*c*_1_,*c*_2_,…,*c*_*i*_}∈[0,1] is an adjustable coefficient that we define as intervention index, of which a higher value implies stricter mobility control. This adjustment is weighted with the mobility reduction affected by the intervention strategies imposed. Practically, this metric can be configured by an intervention function, where time *t*, infection numbers *I* or risk index *R* serves as an independent variable (Table 1). We assume an *i*×*i* traffic volume matrix *V* derived by Mao et al.’s prediction [18] as the passenger estimation in normal cases, whose elements *V*_*mn*_ quantifies the normal traffic volume from node *m* to node *n*. The traffic matrix $\hat{V}$ is estimated by:

$$\hat{V}=AVA$$ (3)

**Table 1:** Comparison of various intervention strategies for mobility simulation.

No.	Strategy	Intervention index configuration	Average traffic volume	Average EPS
(1)	None	*c* = 0	1548.394	2.972
(2)	Constant	$\left\{ \begin{array}{l} c(Hubei)=0.99,\\ c(Beijing,Shanghai,Guangzhou)=0.25,\\ c(others)=0.125 \end{array}\right.$	758.766	1.760
(3)	*t*-Function	$\left\{ \begin{array}{l} c(Hubei)=0.99,\\ c(others)=\max\left\{-\frac{1}{8500}{\left(t-45\right)}^{2}+0.25,0\right\} \end{array}\right.$	759.353	1.669
(4)	*I*-Function	$c=\min\left\{8.25\times 10^{4}\frac{I}{P},1\right\}$	763.821	1.081

**Fig 1:**
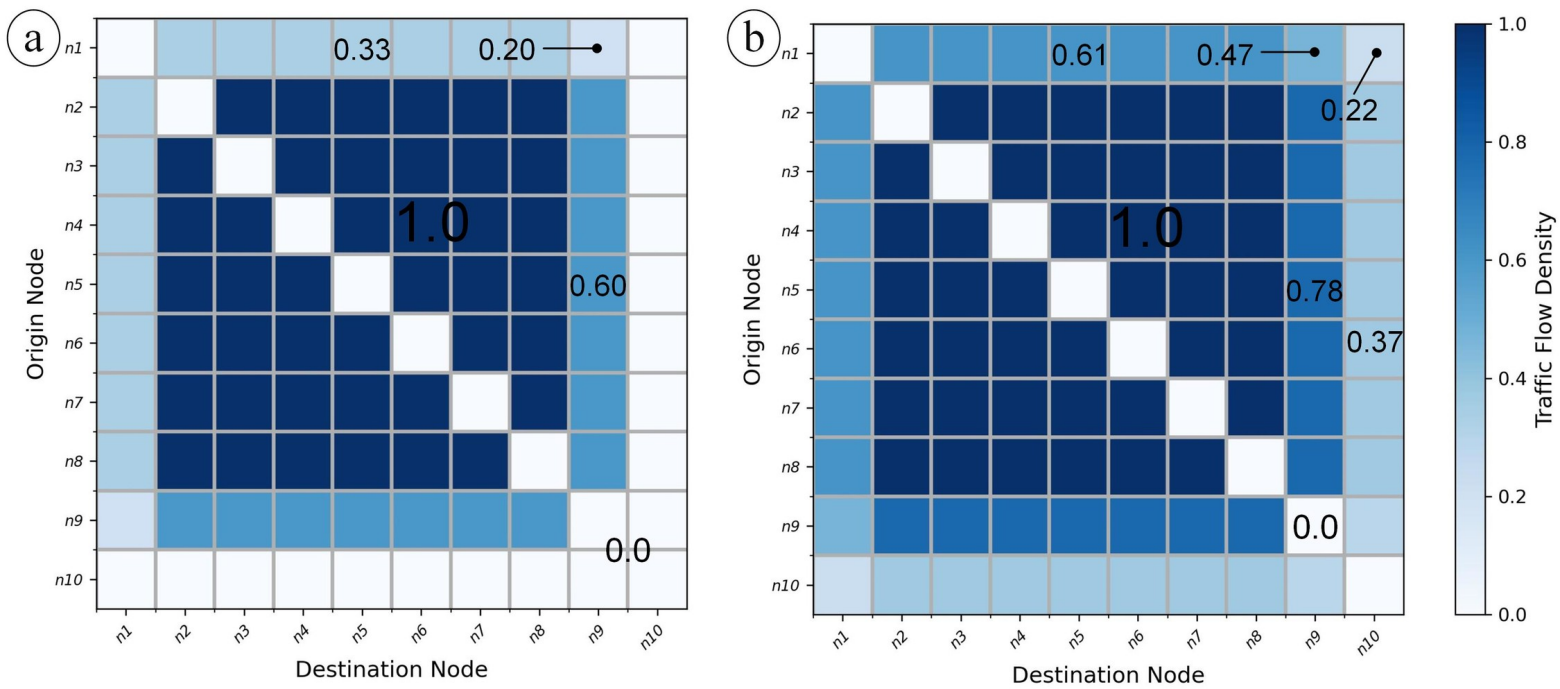
Toy example of how the mobility simulation results are affected by (a) intervention index and (b) risk index. The initial relative flow volume through each pair of nodes is 1.0 (dark blue pixels). After the mobility restriction or risk impact, the traffic densities from or towards the target nodes are reduced and the reduction effect of origin and destination is superimposed (light blue pixels). If an intervention index is fixed to 1, all traffic values export from and import to this node are cut down to 0 (white pixels on node *n*_10_ in Fig 1A), which indicates a complete lockdown.

Through this control, both the export volume and import volume are reduced. For example, given 10 nodes *n*_1_,*n*_2_,…,*n*_10_ to construct a 10×10 origin-destination matrix, each traffic value from one node to another is 1, and the risk index on each node is 0. If we configure the intervention index by setup *S*, where node *n*_1_ is configured as 0.5, *n*_9_ as 0.25, *n*_10_ as 1, and other nodes as 0, we learn how a restriction strategy on these nodes can globally effect the traffic condition (Fig 1A). If we configure intervention index to 0 and risk index by *S*, the global traffic impact of risk index is illustrated (Fig 1B).

### 2.3 Epidemic strength estimation based on effective distance

In terms of evaluating the intervention effect, we propose a concept of Epidemic Strength (EPS) to depict the transmission hazard involved in the mobility network, especially in the edges. The core notion of EPS is to estimate the spread threat from one node to another by measuring the effect through a certain dominant path. To describe the connective strength between each node, we first compute the effective distance *d*_*mn*_ from node *m* to its connected node *n* as follows:

$$d_{mn}=1-\log\frac{{\hat{V}}_{mn}}{{\hat{V}}_{m}}$$ (4)

where ${\hat{V}}_{mn}$ denotes the predicted traffic volume travelled from node *m* to node *n*, ${\hat{V}}_{m}$ denotes the total volume travelled from node *n*. If nodes *m*, *n* are not directly connected, for example, assuming that Γ = {*γ*_1_,*γ*_2_,…,*γ*_*L*_} is a range of possible paths from node *m* to *n*, *λ*(*γ*) is the total effective length along path *γ*, the effective distance *D*_*mn*_ from node *m* to *n* can be computed as:

$$D_{mn}=\underset{\gamma \in \Gamma }{\min}\lambda (\gamma )$$ (5)

Here we use *D*_*mn*_ to denote effective distance from node *m* to *n* if *m*, *n* are not directly connected. It should be noted that *d*_*mn*_≠*d*_*nm*_ and *D*_*mn*_≠*D*_*nm*_, which means the effective distances between two nodes are directionally biased (S2 Fig). After measuring the effective distances between each pair of nodes, we can estimate the matrix of epidemic strength Φ as follows:

$$\Phi =\log\left(\frac{diag(I/P)∙\hat{V}}{D}+J\right)$$ (6)

where *I* is a vector of infected numbers of *i* nodes, *P* indicates a range of populations of corresponding nodes, $\hat{V}$ is the aforementioned *i*×*i* traffic matrix, *D* denotes an *i*×*i* matrix with elements *D*_*mn*_ indicating effective distance from node *m* to node *n*, *J* represents a *i*×*i* matrix of ones. Φ is an *i*×*i* matrix of epidemic strength *φ*. The EPS serves as a strong signal for analyzing spread potentials within a mobility network, where higher *φ* values indicate greater dissemination risks along the routes. This score integrates epidemiological statistics with mobility features to enable an insightful epidemic comprehension. By configuring the intervention index in Eq (2), we can analyze the coronavirus EPS values across the China mobility network and predict the intervention effects under different levels of strategies.

## 3. Results and discussion

**Fig 2:**
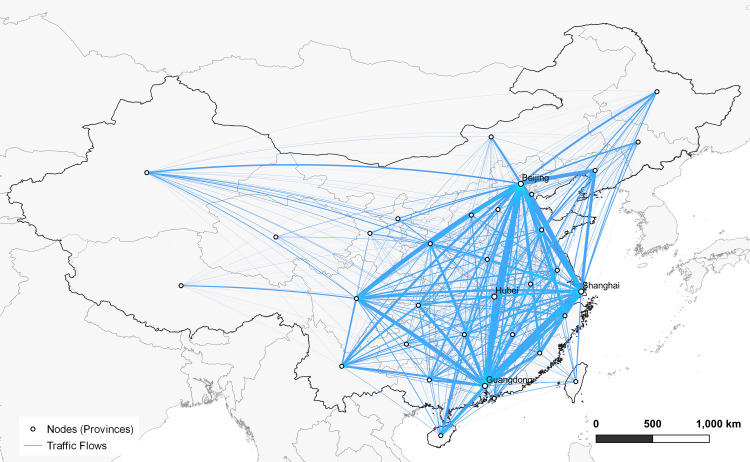
Aggregated mobility network of Chinese provinces. The nodes denote provincial-level administrative divisions and the edges denote aggregated traffic flows between each pair of nodes. The width of each link indicates passenger volume predicted by historical data.

To match the spatial granularity of the statistical dataset of COVID-19 and population, we first aggregate the airline data into a mobility network of provinces. Fig 2 shows the derived network that covers 33 nodes and 264 edges, where the total traveler numbers of normal estimation (without epidemic effects) is depicted by edge thickness. It is clearly observed that China’s geospatial mobility exhibits a southeast-northwest pattern—broadly, the flights across southeast China contain more passenger volume. This suggests that the southern and eastern cities should raise more cautions to counter the virus impact. Besides, the normal passenger data and actual passenger data are collected at monthly granularity. To ensure all the input data meet daily granularity and simulate our results at daily level, we first perform interpolation on these data. From the comparative plot (S3 Fig), we observe that regardless of the coronavirus impact, the normal prediction results exhibit generally higher traffic (blue lines). On the contrary, considering the effect of risk index, our simulation results (green lines) fit better to the actual traffic observation (grey line), which indicates the virus impact on the transportation industry.

**Fig 3:**
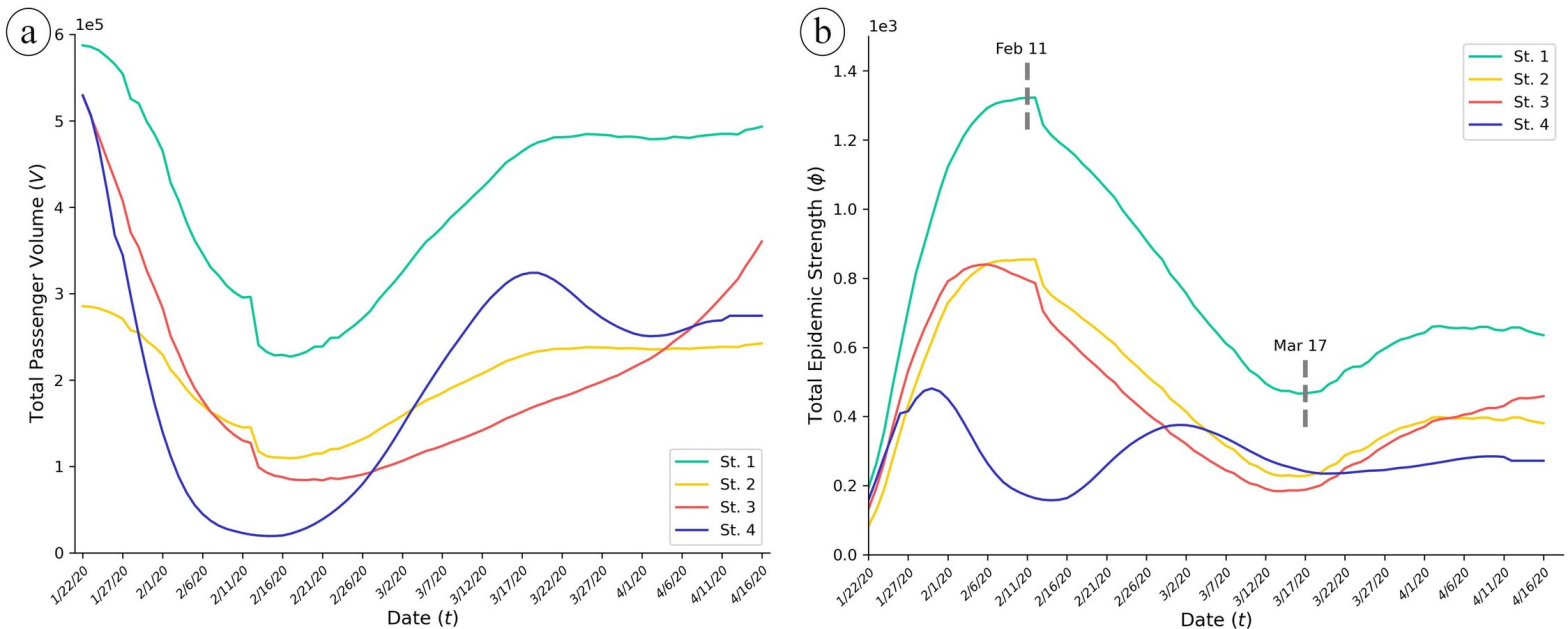
Overview of mobility intervention impacts on COVID-19 transmission control. (a) Total traffic volume (*V*) estimation under different intervention index (*c*) configuration. The overall traffic drops due to the early outbreak and progressively recovers as the increasingly effective intervention yielded lower infection cases. The prediction results with 95% confidence intervals is plotted in S4 Fig. (b) Total epidemic strength (*φ*) estimation under different control strategies, where the EPS values clearly indicate the outbreak severity and mitigation effect.

According to our mobility simulator, we estimate the daily traffic amount with different intervention parameters and then compute the corresponding EPS values. All matrices of traffic volume and risk index are estimated at daily granularity, and the population in each node is assumed to be constant. We formulate four mobility intervention strategies for a comparative analysis. Strategy 1 exerts absolutely no intervention on each node, while strategies 2, 3 and 4 restrict human mobility following different schemes—constant, function of time and function of infection number. We adjust the traffic volume mean obtained by strategies 2, 3, 4 to be approximately equal (Table 1). With no intervention, Strategy 1 has noticed a peak value of EPS on February 11, which indicates a probable maximum number of infections. Comparing to no-response strategy, all the intervention strategies exhibit certain effect against the epidemic transmission (Fig 3B). During the entire epidemic period, Strategy 2 suppresses the flights with a constant intensity, and the passenger volume shows a similar pattern to Strategy 1. Strategy 3 controls the traffic following a parabolic function of time *t*, where the intervention index reaches its peak at the 45th day (March 7). The EPS levels derived from strategies 2 and 3 are roughly the same (Fig 3B). The mobility restriction in Strategy 4 completely follows the ratio of the infection number to the population on each node, which leads to the lowest amount of EPS (Table 1). It is noticed that when the epidemic worsens, the traffic curve under this strategy drops significantly, thus successfully curbing the transmission risk during the critical period. These observations strongly suggests that, when dealing with highly infectious diseases, it would be rational to take more restrictive interventions in severely infected cities or during critical periods and discretionarily loosen the reins when the situation improves. This also provides advice on how to control the virus spread while preserving as much communication as possible.

**Fig 4:**
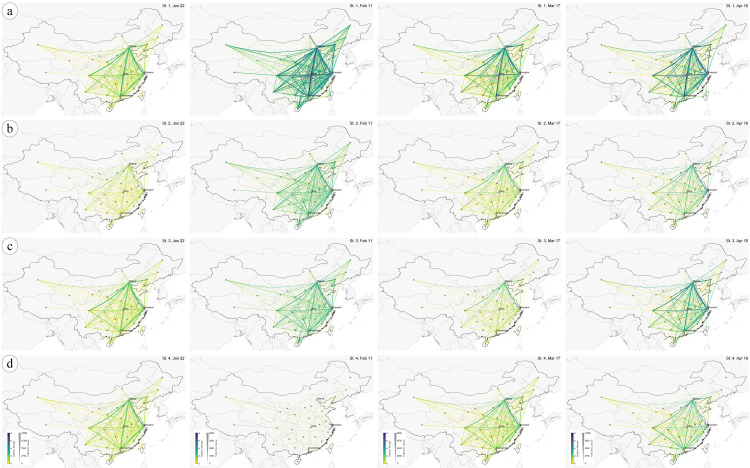
Flow map visualization of epidemic strength evolution following four intervention strategies—(a) Strategy 1, (b) Strategy 2, (c) Strategy 3, and (d) Strategy 4 (Table 1). The edge color indicates epidemic strength and the edge width indicates traffic amount. For better illustration and comparison, each column of snapshots (on the same date) shares the same legend and representation. Without public intervention, Strategy 1 shows the significantly highest risk throughout the period. In other strategies, mobility restrictions result in reduced traffic and hence lower risk of epidemics. Also, constraints on crucial nodes help efficiently reduce the disease damage (interventions on Hubei). Overall, Strategy 4 shows a better epidemic mitigation effect. For a clear and detailed image, please refer to the online version of this paper.

With regard to effective distance, the effective distances and geographic distances between each pair of nodes are rendered in a color-coded image view (S2 Fig), and the correlation between the two distance indicators and disease arrival time (the date of the first confirmed case in each node) are analyzed (S5 Fig). For in-depth insights, we further compute the EPS of each pair of nodes in the context of the four intervention strategies in Table 1. The estimated results cover the period from January 22 to April 16. To create a comprehensive visualization effect, we depict the directional measurements in a flow map, where EPS is rendered as color and traffic volume as line width. We also implement force-directed edge bundling for a clearer display [27]. For each of the intervention strategies, we choose four representative snapshots to display: January 22, February 11, March 17 and April 16 (Fig 4), which correspond to four periods of epidemic evolution—emergence, peak, alleviation and calm.

From a temporal aspect, the overall EPS exhibits a clear, undulating pattern that corresponds to the depiction in Fig 3B. The emergence period observes the lowest EPS of the four periods, where some nodes notice relatively higher EPS values, such as Hubei (highest infection number), Beijing, Shanghai and Guangdong (huge traffic amount). The second period shows the highest EPS values and lowest traffic volumes, especially in Hubei, which suggests that the transmission risk has soared and damaged the traffic condition. The periods of alleviation and calm indicate that the public health is progressively improving and the traffic amount is recovering. During the alleviation period, higher EPS values are observed on Hubei and Beijing. As the epidemic was effectively weakened and certain traffic resumed, the calm period shows a subtle growth in EPS. From a spatial perspective, most of the hazardous nodes and links are located across the southeast. The edges connecting high-risk nodes (Hubei, Beijing, etc.) present high EPS values, especially edges that directly links Hubei. From an intervention point of view, by imposing strong mobility restrictions on key nodes or critical periods, a robust mitigation effect is demonstrated. During the emergence period, Strategy 2 shows the lowest traffic amount but not very notably reduced EPS due to the small number of infections (Fig 4B, snapshot 1). Although fiercest virus spread is observed during the peak period, Strategy 4 has strongly restricted the human mobility and successfully controlled the epidemic strength (Fig 4D, snapshot 2). Comparing Strategy 1 with the others, when certain constraint is imposed on a single node (e.g., Hubei), the transmission threat on all paths that connects this node is considerably reduced, while the global traffic is slightly weakened. Generally speaking, during the entire epidemic, Strategy 4 provides a superior configuration and achieves the healthiest outcome overall (Fig 4D). These results may lend insight into the significance of restricting the mobility of highly hazardous nodes.

## 4. Conclusion

This research proposed a mobility-based approach for analyzing the positive effect of intervention strategies when facing highly prevalent pandemics like COVID-19. Due to the complexity of communication, accurate epidemic dynamics are usually unpredictable. Our method tackled the problem from a human-mobility perspective and developed a simulation analytics. In the case study of China mobility network, our quantitative estimation of epidemic strength demonstrated that during the early stage of COVID-19, travel restriction strategies can effectively reduce the transmission risks through the mobility network. Our study only considered air traffic mobility data, while some other modes of transportation (such as rail or road) also transport passengers between domestic cities. In this concern, more extensive research that covers various types of mobility data is expected in future studies. From the analysis, China’s major transmission threat is noticed at the southeast region, which seems to be correlated with a range of economic, technological or social factors. More future works are expected in evaluating the impact of these factors. Further, combined with bidirectional graph, this approach is expected to solve more meaningful problems, which we hope can provide insights for future researchers.

## Supporting information

S1 Fig China airline network for human mobility representation. (TIF)

S2 Fig Understanding effective distance through an image visualization, where the Y-axis indicates origin nodes and the X-axis indicates destination nodes. (a) Province-to-province effective distance view. Lower values of effective path (e.g., Beijing and Shanghai) suggest higher potential risks. Differences can be observed between same nodes but different directions. (b) Province-to-province geographic distance view.
(TIF)

S3 Fig Comparison of total passenger numbers estimated by various approaches. The grey line indicates the real traffic observation, the blue lines represent Mao et al.’s prediction results and prediction bands, and the green lines and green-shaded region indicate our simulation results and confidence bands without intervention (i.e., Strategy 1 in Table 1, *R*^2^ = 0.925). Due to possible missing data, the simulation results do not include every flight data and may not perfectly match the observation curve. Nevertheless, compared with the normal prediction results, the simulation results exhibit a more similar temporal pattern to the observation results (MSE(*St*.1) = 0.013, MSE(*Mao*′*s*) = 0.072).
(PDF)

S4 Fig Total traffic volume (*V*) estimation results in Fig 3A with 95% confidence intervals. (PDF)

S5 Fig Understanding the effective distance as a signal for contagious events. (a) Arrival time versus effective distance for each of the 33 nodes in the mobility network (Fig 2). The size of each dot indicates the total airline routes through the node. (b) Arrival time versus geographic distance for each node. The effective distance exhibits a much higher correlation with arrival time (*R*^2^ = 0.705) than geographic distance (*R*^2^ = 0.375).
(TIF)
