# Supplementary figures and images for: Modeling and interpreting the COVID-19 intervention strategy of China: A human mobility view
Source: PLoS One. 2020 Nov 24;15(11):e0242761. doi: 10.1371/journal.pone.0242761 (PMC7685462; doi:10.1371/journal.pone.0242761)

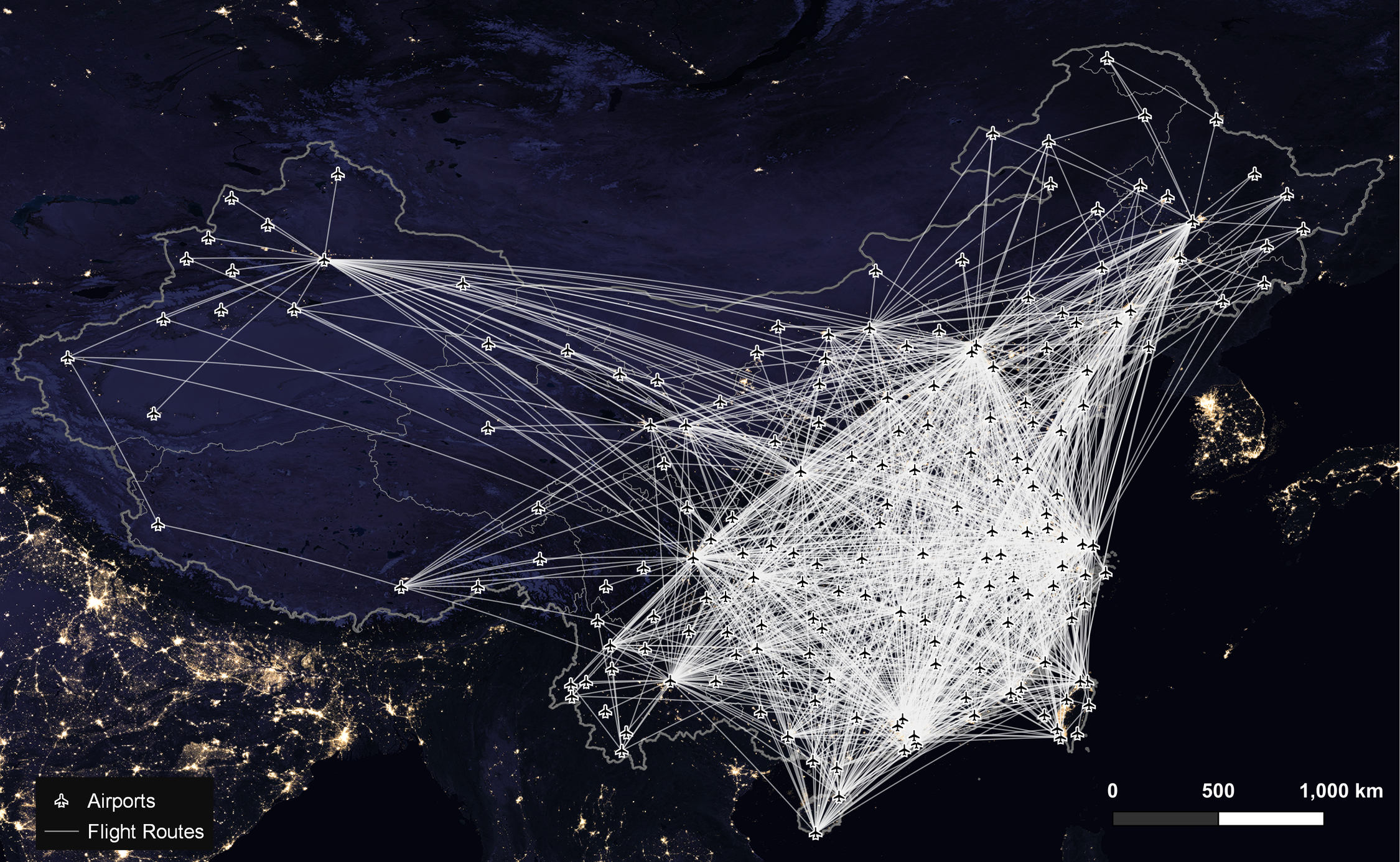

Supplement: S1 Fig — (TIF) [file pone.0242761.s001.tif]

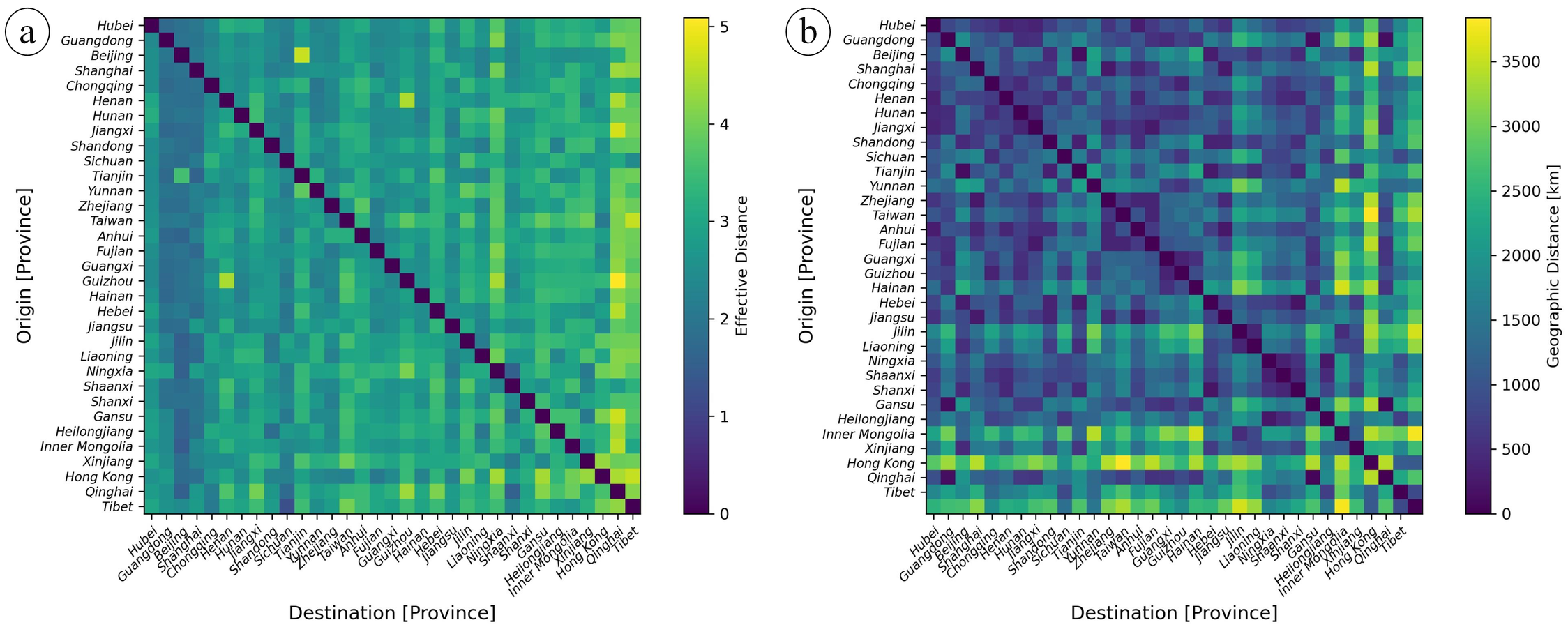

Supplement: S2 Fig — (a) Province-to-province effective distance view. Lower values of effective path (e.g., Beijing and Shanghai) suggest higher potential risks. Differences can be observed between same nodes but different directions. (b) Province-to-province geographic distance view. (TIF) [file pone.0242761.s002.tif]

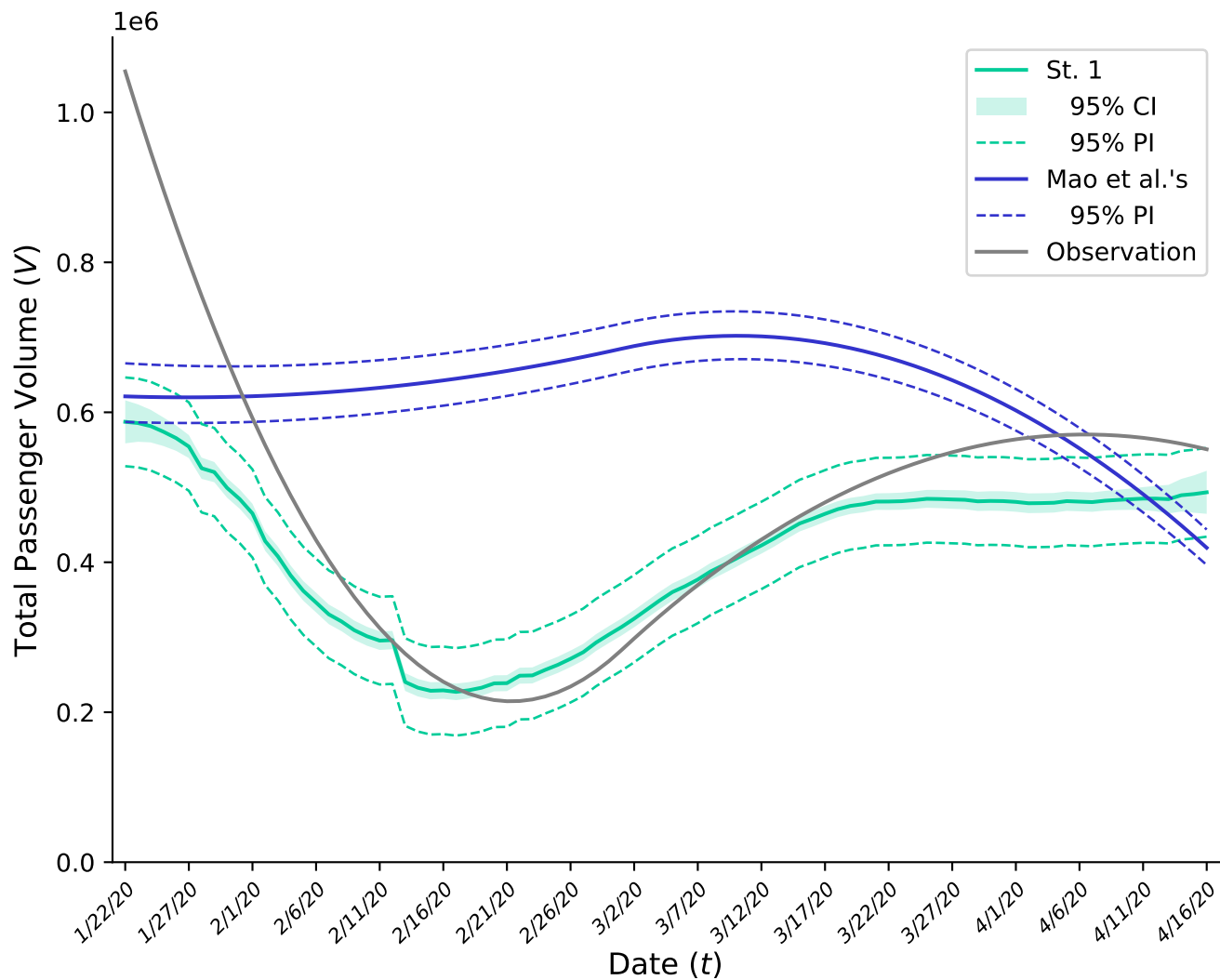

Supplement: S3 Fig — The grey line indicates the real traffic observation, the blue lines represent Mao et al.’s prediction results and prediction bands, and the green lines and green-shaded region indicate our simulation results and confidence bands without intervention (i.e., Strategy 1 in Table 1, R2 = 0.925). Due to possible missing data, the simulation results do not include every flight data and may not perfectly match the observation curve. Nevertheless, compared with the normal prediction results, the simulation results exhibit a more similar temporal pattern to the observation results (MSE(St.1) = 0.013, MSE(Mao′s) = 0.072). (PDF) [file pone.0242761.s003.pdf]

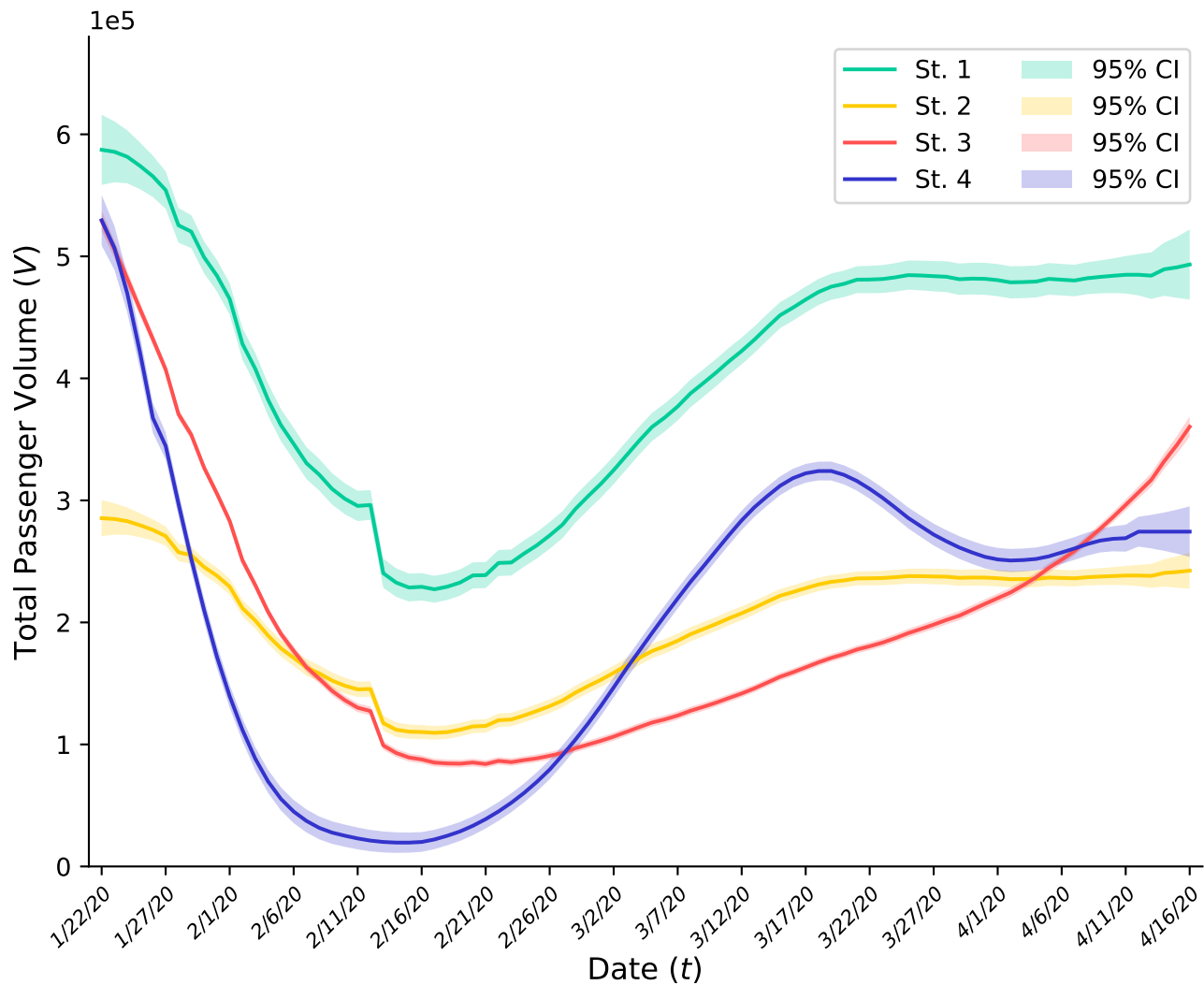

Supplement: S4 Fig — (PDF) [file pone.0242761.s004.pdf]

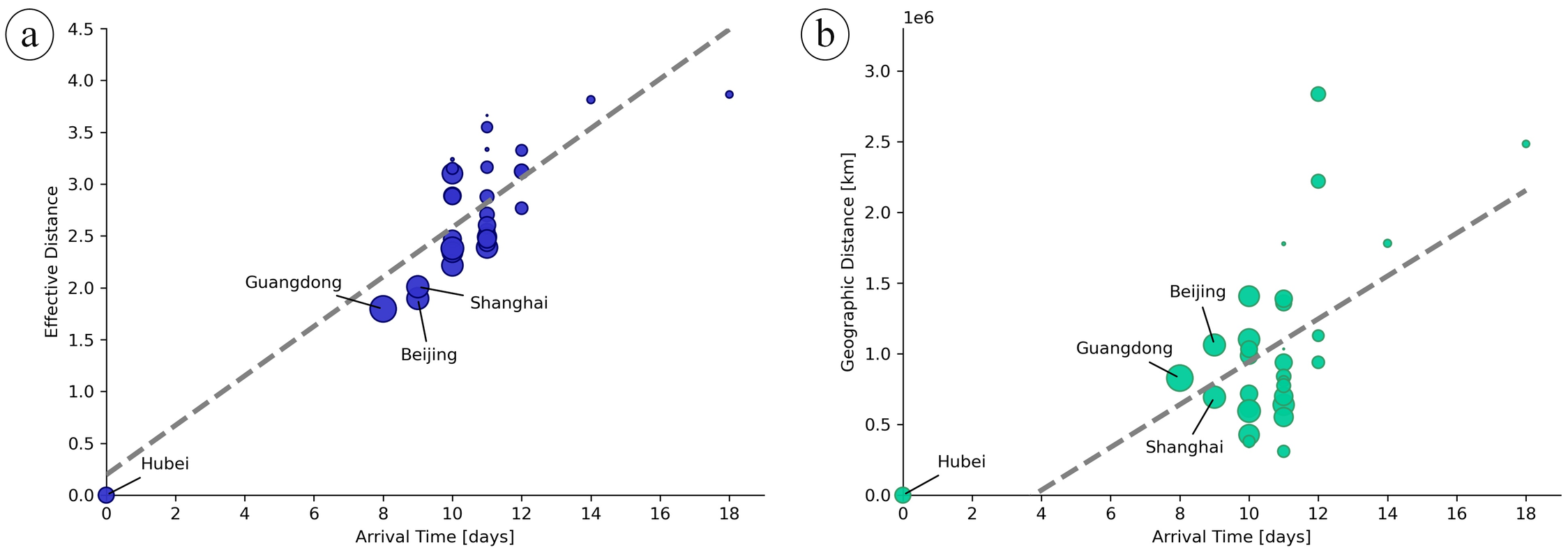

Supplement: S5 Fig — (a) Arrival time versus effective distance for each of the 33 nodes in the mobility network (Fig 2). The size of each dot indicates the total airline routes through the node. (b) Arrival time versus geographic distance for each node. The effective distance exhibits a much higher correlation with arrival time (R2 = 0.705) than geographic distance (R2 = 0.375). (TIF) [file pone.0242761.s005.tif]
